# 

**Published:** 1903-09

**Authors:** 


### CONTENTS.

ORIGINAL COMMUNICATIONS—

Some Social Factors That Tend to the Degeneration of the Race and Alcoholism. By R. R. Kime, M.D., Atlanta, Ga. .......... 361

Chairman's Address. By Dunbar Roy, M.D., Atlanta, Ga. .......... 372

The Status of the Drug Business in Atlanta. By Samuel A. Visanska, Ph.G., M.D. .......... 378

The International Quarantine Bureau. By J. M. Lindsley, M.D. .......... 382

EDITORIAL NOTES AND COMMENTS—

A State Board of Health .......... 385

The Odor of the White Race .......... 388

Atlanta's Milk Supply .......... 390

NEWS AND NOTES .......... 391

CONTENTS CONTINUED .......... x

